# Targeting of CYP17A1 Lyase by VT-464 Inhibits Adrenal and Intratumoral Androgen Biosynthesis and Tumor Growth of Castration Resistant Prostate Cancer

**DOI:** 10.1038/srep35354

**Published:** 2016-10-17

**Authors:** Sankar N. Maity, Mark A. Titus, Revekka Gyftaki, Guanglin Wu, Jing-Fang Lu, S. Ramachandran, Elsa M. Li-Ning-Tapia, Christopher J. Logothetis, John C. Araujo, Eleni Efstathiou

**Affiliations:** 1 Department of Genitourinary Medical Oncology, David H. Koch Center for Applied Research of Genitourinary Cancers, The University of Texas MD Anderson Cancer Center, Houston, Texas; 2 Department of Oncology, Alexandra Hospital, University of Athens, Athens, Greece

## Abstract

Cytochrome P450 17α-hydroxylase/17,20-lyase (CYP17A1) is a validated treatment target for the treatment of metastatic castration-resistant prostate cancer (CRPC). Abiraterone acetate (AA) inhibits both 17α-hydroxylase (hydroxylase) and 17,20-lyase (lyase) reactions catalyzed by CYP17A1 and thus depletes androgen biosynthesis. However, coadministration of prednisone is required to suppress the mineralocorticoid excess and cortisol depletion that result from hydroxylase inhibition. VT-464, a nonsteroidal small molecule, selectively inhibits CYP17A1 lyase and therefore does not require prednisone supplementation. Administration of VT-464 in a metastatic CRPC patient presenting with high tumoral expression of both androgen receptor (AR) and CYP17A1, showed significant reduction in the level of both dehydroepiandrosterone (DHEA) and serum PSA. Treatment of a CRPC patient-derived xenograft, MDA-PCa-133 expressing H874Y AR mutant with VT-464, reduced the increase in tumor volume in castrate male mice more than twice as much as the vehicle (P < 0.05). Mass spectrometry analysis of post-treatment xenograft tumor tissues showed that VT-464 significantly decreased intratumoral androgens but not cortisol. VT-464 also reduced AR signaling more effectively than abiraterone in cultured PCa cells expressing T877A AR mutant. Collectively, this study suggests that VT-464 therapy can effectively treat CRPC and be used in precision medicine based on androgen receptor mutation status.

Cytochrome P450 17α-hydroxylase/17,20-lyase (CYP17A1), a key enzyme for sex steroid biosynthesis expressed primarily in the testes and adrenal gland, is a validated target for treatment of metastatic castration-resistant prostate cancer (CRPC). Recent experimental evidence in preclinical and clinical settings has demonstrated that expression of both androgen receptor (AR) and CYP17A1 predicts the active intracrine androgen signaling that drives CRPC^1^,^2^. CYP17A1 is dual-function enzyme that, through its 17α-hydroxylase (hydroxylase) activity, adds a hydroxyl group to the 21-carbon steroid precursors pregnenolone and progesterone and then utilizes its 17,20-lyase (lyase) activity to cleave 17α-hydroxypregnenolone to dehydroepiandrosterone (DHEA), the androgen precursor of testosterone and dihydrotestosterone (DHT). CYP17A1 lyase represents the first committed step in androgen biosynthesis (Fig. 1A) in all steroidogenic tissue as well as in the adrenal. Both testosterone and DHEA can be converted into potent androgen DHT in prostate^3^. Published study demonstrated that CRPC tumors contain key steroidogenic enzymes including CYP17A1 that can drive intratumoral de novo steroid biosynthesis^4^. Medical castration treatment inhibits production of testicular testosterone but does not impact production of adrenal DHEA or intratumoral androgen, which can be responsible for driving CRPC. The CYP17A1 hydroxylase activity, but not the lyase activity, is also needed for biosynthesis of glucocorticoids in the adrenal, since glucocorticoids are derived from 17α-hydroxyprogesterone^3^.

Abiraterone acetate (AA; Zytiga) in combination with prednisone is approved for treatment of CRPC. AA specifically and irreversibly inhibits both CYP17A1 hydroxylase and lyase^5^. AA-prednisone therapy demonstrated a 4-month survival benefit compared to placebo as well as quality-of-life benefits and improvements in skeletal-related events for CRPC patients^1^,^2^,^6^,^7^. However, inhibition of 17α-hydroxylase by AA depletes the adrenal biosynthesis of glucocorticoids, which induces an increase in adrenocorticotropic hormone from the anterior pituitary gland thus producing side effects that are only partially suppressed by co-administration of the cortisol replacement prednisone^7^. Overall, CYP17A1 inhibition provides significant benefits to CRPC patients due to suppression of androgen signaling, which stimulates prostate tumor growth.

A selective inhibitor of CYP17A1 lyase has the potential to improve the side effect profile of the AA-prednisone therapy. In this study, we investigated VT-464, a small-molecule, CYP17A1 inhibitor that, as recently reported, was optimized for lyase selectivity and oral activity^8^. In contrast to abiraterone, VT-464 is nonsteroidal and utilizes a less avid 1,2,3-triazole compared to the tight-binding pyridine in abiraterone (Fig. 1B). The lower-avidity 1,2,3-triazole moiety contributes lyase selectivity to VT-464. The *in vitro* CYP17A1 enzyme assay showed that VT-464 lyase inhibition was 10-fold as potent as hydroxylase inhibition; in contrast, abiraterone inhibited hydroxylase 6-fold more potently than lyase^8^.

The objective of the current study was to assess the effects of VT-464 on a CRPC patient as well as two different experimental models, in a CRPC patient-derived xenograft (PDX) tumor in mice and in a prostate cancer cell line in culture. In the model experiments, the effects of VT-464 were compared to those of abiraterone or its orally available acetate form.

## Results

### Impact of VT-464 on a CRPC patient

**Figure 2:**
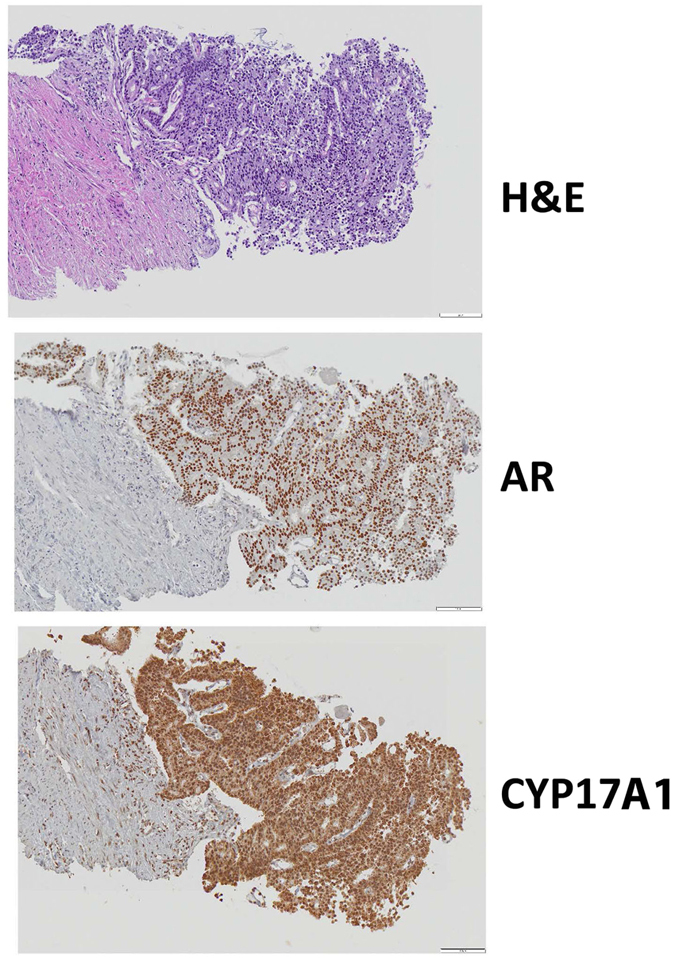
Representative images of histologic analysis of patient needle core prostate biopsy collected before treatment. (**A**) Hematoxylin and eosin stain (H&E) showing high-grade adenocarcinoma at the tip (right side) and adjacent stroma (left side). Immunohistochemistry of the same area showing (**B**) strong, diffuse expression of androgen receptor detected by N-terminal AR antibody, and (**C**) strong diffuse expression of CYP17A1. Original magnification, X200.

**Figure 3:**
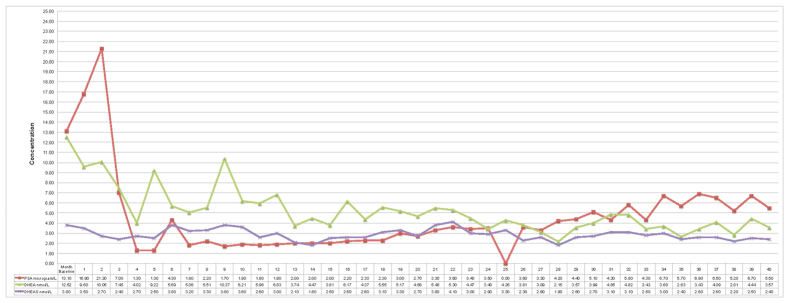
Levels of PSA, DHEA, and DHEAS in patient plasma during treatment with VT-464 collected at baseline and each month for 40 months. Normal levels of DHEA and DHEA sulfate in men 70 to 80 years old range from 4 to 5.3 nM and 1330 to 1750 nM, respectively^34^,^35^.

Prostate biopsies identified adenocarcinoma with a Gleason score 8 (4 + 4) in the left peripheral prostate lobe of a 68 year old man in 2002. The patient was treated with LHRH antagonist, Leuprolide, and androgen receptor (AR) antagonist Bicalutamide to January 2012 followed by 3 months of somatostatin analog, Lanreotide, due to rising PSA. Computed tomography scans confirmed metastasis to bone and VT-464 therapy (300 mg twice a day) without prednisone was initiated. Immunohistochemistry analysis of the prostate biopsies showed expression of high level of both AR and CYP17A1 (Fig. 2). Measurement of plasma PSA, ACTH, and steroid profile were performed at baseline (May 2012) and each month during treatment with VT-464 from June 2012 to August 2015. Both PSA and adrenal androgen, dehydroepiandrosterone (DHEA), levels were decreased by VT-464 within a few months of the treatment (Fig. 3). The DHEA level remained low during the treatment duration. However, after 30 months of treatment, the PSA level started to rise indicating development of resistant tumors. Testosterone and androstenedione levels were very low due to Leuprolide and were unchanged during the treatment (Supplementary Figure 1). The cortisol level was unaffected by the treatment indicating VT-464 selectively did not inhibit CYP17A1 hydroxylase activity (Supplementary Figure 2). Levels of progesterone and corticosterone were also unchanged by the treatment.

### Effects of VT-464 and AA on CRPC patient tumor derived xenograft (PDX) tumors in mice

The impact of oral VT-464 and AA on growth of MDA-PCa-133 prostate cancer PDX tumors was determined in castrate mice. The MDA-PCa-133 PDX tumor, which expresses an AR with a H874Y mutation within the ligand-binding domain and secretes PSA, was initially established and maintained as subcutaneous tumors in noncastrated SCID male mice. At passage 5, the xenograft tumor tissue was implanted subcutaneously in castrate male SCID mice, in which the tumor was established and maintained. The rates of tumor growth and serum PSA increases were very similar in noncastrate and castrate mice (Supplementary Figure 3). Expression analysis of total tumor RNAs showed that expression of both the full-length AR and AR-V7 were significantly higher in castrate mouse tumors than in noncastrate mouse tumors (Supplementary Figures 4A,B). Immunoblot analysis of protein lysates showed an increase in AR and AR-V7 and also an increase in CYP17A1 protein in tumors from castrate mice (Supplementary Fig. 4C). Expression of AR was also analyzed by immunohistochemistry, which showed increased AR expression in both the nucleus and cytosol of tumor cells grown in castrate mice (Supplementary Figure 4D). Measurement of intratumoral testosterone levels using liquid chromatography–triple quadrupole mass spectrometry (LC/MSMS) showed that the tumors grown in castrate mice had 5-fold higher concentrations than tumors grown in noncastrate mice (0.1 ng/g and 0.02 ng/g of tissue, respectively) (Supplementary Table 1). In contrast, liver testosterone concentrations in tumor-bearing castrate mice were significantly lower than in tumor-bearing noncastrate mice (<0.001 and 0.01 ng/g of tissue, respectively). Altogether, the results indicate that MDA-PCa-133 tumor growth in castrate mice was due to increased expression of AR and its ligand-independent isoform AR-V7, as well as CYP17A1, which is required for intratumoral testosterone biosynthesis.

**Figure 4:**
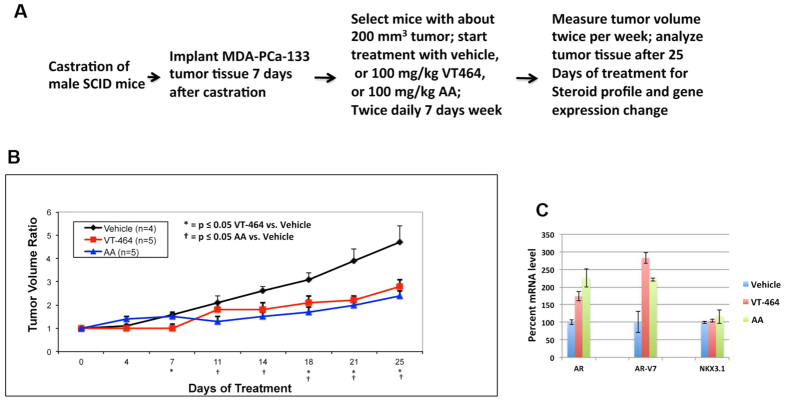
Impact of VT-464 and AA treatment on MDA-PCa-133 tumor growth in castrate SCID mice. (**A**) Tumor treatment schedule; (**B**) tumor volume ratio change during oral treatment with VT-464 or AA; (**C**) expression analysis by QRT-PCR after 25 days of treatment. The P values for differences in expression for VT-464 vs. vehicle and for AA vs. vehicle, respectively, are as follows: *AR*, P < 0.0032 and P < 0.004; *AR-V7*, P < 0.0017 and P < 0.008; *NKX3.1*, P < 0.46 and P < 0.45.

Castrate mice bearing MDA-PCa-133 tumors were treated with oral VT-464, AA, or vehicle for 28 days. Oral VT-464 or AA at 100 mg/kg twice-daily significantly reduced tumor growth (P < 0.05) (Fig. 4A,B). Analysis of posttreatment tumor tissues by LC/MSMS showed that VT-464 and AA decreased intratumoral testosterone levels by 65.2% (P = 0.04) and 60.8% (P = 0.03), respectively, and decreased DHT levels by 82.9% (P = 0.04) and 80.4% (P = 0.03), respectively (Table 1). This indicates that both CYP17A1 inhibitors depleted production of intratumoral androgen. Importantly, intratumoral cortisol also decreased significantly in response to AA but not VT-464 (Supplementary Table 2 and Supplementary Figure 5), indicating that VT-464 did not impact CYP17A1 hydroxylase activity. This result demonstrated specific inhibition of the CYP17A1 lyase by VT-464, associated with inhibition of tumor growth in castrated mice. Our analysis also showed that VT-464 and AA increased expression of full-length AR mRNA 1.7-fold (P < 0.0032) and 2.25-fold (P < 0.004), respectively, and increased AR-V7 mRNA 2.8-fold (P < 0.0017) and 2.2-fold (P < 0.008), respectively (Fig. 4C). In contrast, VT-464 or AA treatment did not affect mRNA expression of the AR signaling gene *NKX3.1*. This indicates the induction of adaptive androgen signaling due to depletion of intratumoral androgen, as previously described^9^,^10^, may be responsible for the remaining MDA-PCa-133 tumor growth and for maintenance of AR signaling in castrate mice.

### Impact of VT-464 on AR signaling in prostate cancer cells

**Figure 5:**
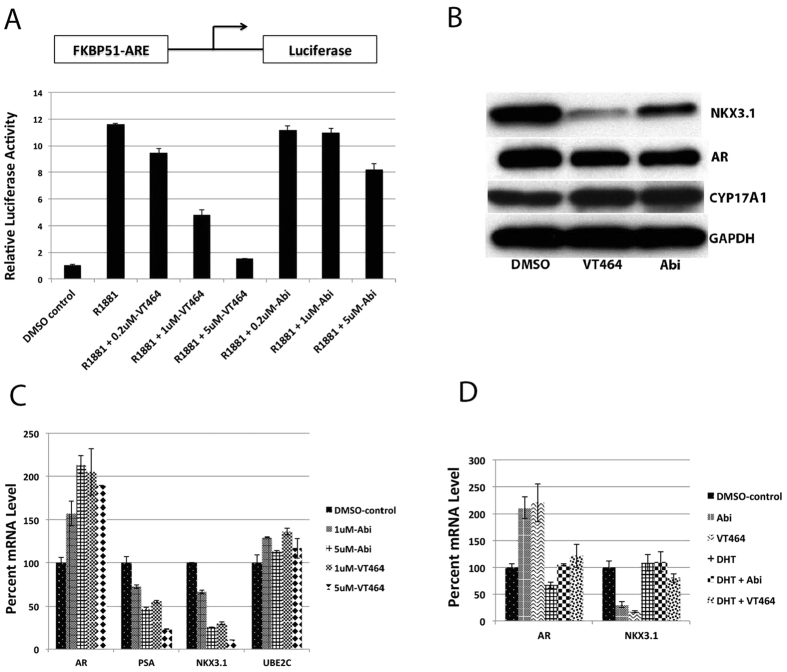
VT-464 inhibited androgen signaling in C4-2B prostate cancer cells. (**A**) The cells were transiently transfected with a luciferase construct under control of FKBP51-ARE promoter and then treated with various concentrations of VT-464 or abiraterone in presence of 1 nM R1881. At 48 h after transfection, luciferase activity in total cell extracts was measured and expressed as fold change of activity for vehicle control (dimethyl sulfoxide; DMSO). (**B**) Expression levels of NKX3.1, AR, CYP17A1, and GAPDH (internal control) proteins were measured by immunoblot in C4-2B cells with or without 5 μM VT-464 or abiraterone. The four gels were run under the same experimental condition, and the individual immunoblots are shown at end of the supplementary information. (**C**) Effects of VT-464 or abiraterone on *AR*, *PSA*, *NKX3.1*, and *UBE2C* mRNA levels in C4-2B cells. (**D**) Effects of 5 μM VT-464 or abiraterone in the presence or absence of 10 nM DHT on *AR* and *NKX3.1* mRNA levels in C4-2B cells.

To determine the impact of VT-464 on AR signaling, we measured the effects of VT-464 on a AR reporter construct, FKBP51-ARE-luciferase, which is activated in the presence of the synthetic androgen 1nM R1881 in C4-2B prostate cancer cells (Fig. 5A). For comparison, the cells were also treated with the same concentrations of abiraterone. The experiment showed that the relative luciferase reporter activity was highly inhibited with increasing amount of VT-464 but modestly by abiraterone. The reporter activity was inhibited by 90% at 5 μM VT-464 but only 30% at 5 μM abiraterone. The results indicated that the androgen-dependent AR reporter activity was inhibited to a greater extent by VT-464 than by abiraterone.

The impact of VT-464 on cellular AR signaling protein markers in C4-2B cells was measured by immunoblot analysis (Fig. 5B). This showed that NKX3.1 protein expression was significantly decreased by VT-464 and abiraterone but expression of CYP17A1 and AR was not significantly affected. In addition, the expression levels of the AR, prostate-specific antigen (PSA [KLK3]), NKX3.1, and UBE2C genes were quantified in C4-2B cells by quantitative real-time PCR (QRT-PCR) (Fig. 5C). VT-464 treatment inhibited expression of *PSA* and *NKX3.1*, stimulated expression of *AR*, and did not affect expression of *UBE2C*. Abiraterone treatment at the same concentrations resulted in very similar effects, except that VT-464 treatment displayed a stronger inhibition of PSA and NKX3.1 gene expression. These experiments showed that VT-464 treatment specifically inhibited AR-stimulated PSA and NKX3.1 gene expression in C4-2B cells. The inhibition of NKX3.1 gene expression by 5 μΜ VT-464 or 5  μΜ abiraterone was abrogated by the addition of 10 nM DHT, demonstrating that the direct or indirect (resulting from the inhibition of androgen biosynthesis) AR-inhibitory effects of VT-464 that lead to *NKX3.1* suppression are negated by exogenous DHT (Fig. 5D). The presence of exogenous DHT prevented abiraterone-induced inhibition of AR-stimulated transcripts more effectively than that due to VT-464.

**Figure 6:**
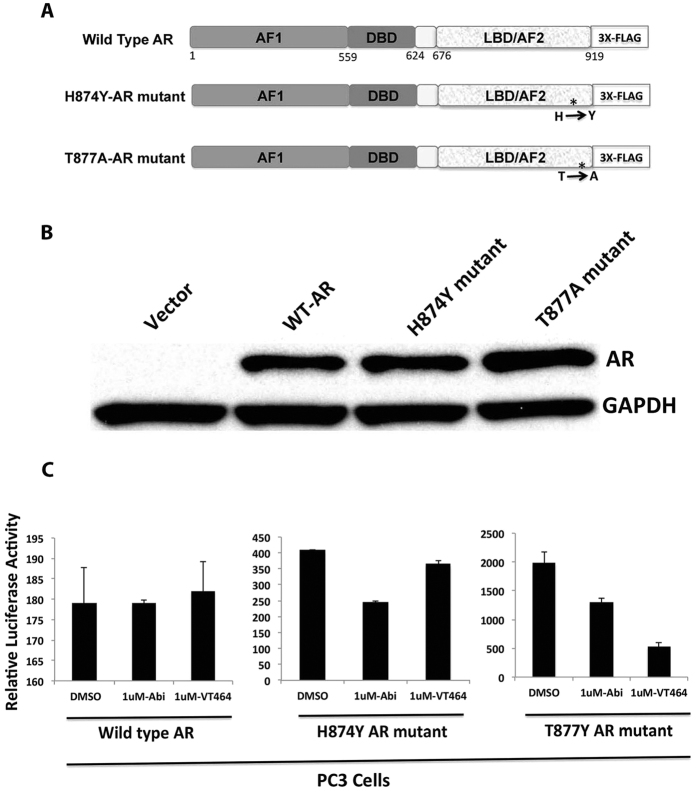
Effects of VT-464 and abiraterone (Abi) as AR antagonists in PC3 prostate cancer cells. Expression of wild-type (WT) and mutant AR expression constructs (**A**) was measured by immunoblot with anti-AR antibody (**B**) after transient transfection in PC3 cells. (**C**) Effects of wild type and mutant ARs on FKBP51-ARE reporter in PC3 cells treated with vehicle, 1 μM abiraterone or VT-464. DBD, DNA binding domain; LBD, ligand-binding domain; DMSO, dimethyl sulfoxide.

We next examined the effects of VT-464 and abiraterone on the AR reporter (FKBP51-ARE-luciferase) activity with overexpression of wild-type AR, two mutant ARs, (Fig. 6A,B) and AR-V7 (Supplementary Figure 6) in AR-negative PC3 prostate cancer cells. Each recombinant protein was expressed as a fusion with a 3x-Flag tag and was detected by the N-terminal AR antibody (Fig. 6B and Supplementary Figure 6). The relative luciferase activity in presence of AR or AR mutants was calculated after subtraction of the basal luciferase activity with the vector plasmid control. Thus the activity in DMSO control (without VT-464 or Abi treatment) is due to specific activation of the ARE reporter by AR or AR mutants expressing in PC3 cells (Fig 6C). The T877A AR mutant substantially increased reporter activity using alternate AR ligands^11^,^12^ and possible stabilization of mutant AR conformation at low ligand levels^13^. Notably, both 1 μM abiraterone and 1 μM VT-464 inhibited T877A AR reporter transactivation by 33 and 75%, respectively (Fig. 6C). In contrast, wild-type AR and mutant H874Y reporter transactivation activity showed no inhibition in the presence of 1 μM VT-464. The H874Y AR mutant activity, which can be regulated by promiscuous ligand binding^11^ was slightly inhibited by 1 μM abiraterone. Expression of AR-V7 stimulated reporter activity 35-fold, and treatment of cells with either VT-464 or abiraterone had no significant impact on AR-V7-dependent reporter activity in PC3 cells (Supplementary Figure 6).

## Discussion

It was previously shown that the oral, nonsteroidal, small-molecule CYP17 lyase inhibitor VT-464, reduced circulating androgens in animal models thereby not promoting the steroid changes that can increase AR signaling and that contribute to abiraterone resistance, without disrupting the mineralocorticoid or glucocorticoid steroidogenic pathways^8^.

Analysis of blood plasma of VT-464 treated patient demonstrated VT-464 effectively inhibited adrenal androgen production but not cortisol during entire 40 months of treatment. The treatment also decreased PSA for 30 months but the last 10 months of treatment a slight increase in PSA levels was observed, indicating development of treatment resistant tumors. Because of very limited amount biopsy samples, we were unable to analyze status of AR or its variants. Thus we analyzed effectiveness of VT-464 in two different CRPC models.

In agreement with other studies of patient-derived xenografts, MDA-PCa-133 displayed similar complex tumor adaptation characteristics of those that occur in the absence of circulating testosterone, including increased expression of CYP17A1, wild-type full length AR and its ligand-independent variant AR-V7, and a gain-of-function mutation in the full-length AR ligand-binding domain sequence (H874Y)^10^,^14^,^15^. MDA-PCa-133 tumors grown in castrate mice also showed increased concentrations of testosterone and DHT, indicating that tumor growth is at least partially hormonally driven. Both oral VT-464 and AA inhibited tumor growth and significantly decreased intratumoral testosterone and DHT in this model. Oral VT-464 or AA also stimulated increased expression of full-length AR and AR-V7, suggesting that both CYP17A1 inhibitors may activate similar tumor adaptation mechanisms. Intratumoral steroid analysis following oral treatments demonstrated that VT-464 selectively reduced concentrations of testosterone and DHT but not cortisol, whereas AA nonselectively decreased testosterone, DHT, and cortisol, confirming VT-464 CYP17A1 lyase selectivity *in vivo*.

VT-464 strongly inhibited androgen-dependent reporter activity in C4-2B cells, which express a full-length AR that harbors the gain-of-function mutation T877A within the ligand-binding domain. The increased inhibition of the luciferase reporter by VT-464 compared to abiraterone was observed in the presence of the synthetic androgen R1881. Both VT-464 and abiraterone also inhibited the expression of the AR-mediated transcripts PSA and NKX3.1 as well as NKX3.1 protein and stimulated transcription of the AR gene. Neither VT-464 nor abiraterone altered the expression of UBE2C, which is activated by AR in a ligand-independent manner^16^ in prostate cancer cells, verifying that VT-464 and abiraterone specifically inhibited ligand-dependent AR functions in C4-2B cells.

The stimulation of AR gene expression by VT-464 or abiraterone is likely due to derepression of the AR gene at a lower level of androgen in C4-2B cells. Similarly, addition of DHT repressed AR gene expression in C4-2B cells. A previous report identified an androgen-dependent enhancer located in the second intron of the AR gene, which mediates repression of AR gene expression at a higher level of androgen through recruitment of the lysine-specific demethylase 1 repressor complex^17^. At a castration level of androgen, the repressor complex is no longer recruited, resulting in stimulation of AR gene expression. This mechanism can also explain the increased expression of AR and AR-V7 in the MDA-PCa-133 tumor model due to castration or treatment with VT-464 or AA.

The intracellular suppression of exogenous DHT stimulation in C4-2B cells strongly suggests that VT-464 had AR antagonist activity. A recent published study compared the AR antagonist and agonist effects of VT-464 to those of abiraterone and the nonsteroidal inhibitor TAK-700^18^. In the AR antagonist assay, the potency of VT-464 was similar to that of abiraterone, demonstrating that AR affinity was consistent with literature reports^19^,^20^.

The same medicinal chemistry rationale that was applied to the design of VT-464 as a selective CYP17A1 lyase inhibitor can be used to explain the wild-type AR and mutant AR binding activity of VT-464^8^.

The AR-dependent reporter activity in PC3 cells, which does not express AR was stimulated by transient overexpression of wild-type AR and two mutant full length AR with respect to the vector plasmid control. This analysis at best can compare the effects of Abi and VT-464 with each other and the three AR receptors. The activation of reporter activity by wild-type AR or H874Y mutant AR was not inhibited by VT-464. The activity of H874Y is modestly inhibited by abiraterone. In contrast, the activity of the T877A mutant was inhibited more potently by VT-464 than by abiraterone, suggesting VT-464 antagonist selectivity for the T877A AR mutant. The relative activity in DMSO control (without VT-464 or Abi treatment) in the three assays differs possibly due to differential activation of the ARE reporter by AR or AR mutants.

Activation of the reporter by the H874Y and T877A AR could occur possibly due to activation by intracellular promiscuous ligands, as noted previously^11^,^19^,^21^. The T877A activation of the reporter was much greater than that due to H874Y, in agreement with the observation that T877A is a gain-of-function AR mutation associated with constitutive nuclear localization^13^. The more effective inhibition of the T877A mutant by VT-464 in PC3 cells is consistent with VT-464 inhibition of AR signaling in C4-2B cells, which express the T877A AR mutant. Since a CRPC phenotype is associated with expression of the AR variant AR-V7, which activates AR signaling in the absence of ligand^15^,^22^,^23^, we examined the impact of VT-464 on AR-V7-mediated reporter activity in PC3 cells. AR-V7 strongly activated the AR-dependent reporter, which however, was not inhibited by VT-464. These results suggest that the AR mutation or variant status could determine the effectiveness of VT-464 treatment of CRPC.

Altogether, our clinical and preclinical study provided insight into the effect of VT-464 on CRPC tumor growth, the steroid metabolome, and androgen signaling.

## Methods

### Chemicals

Abiraterone and AA were prepared at Viamet Pharmaceuticals as described previously^24^,^25^. VT-464 was provided by Viamet Pharmaceuticals^8^.

### Cell lines and transfection

PC3 prostate cancer cells were obtained from the American Type Culture Collection, and C4-2B prostate cancer cells were obtained from The University of Texas MD Anderson Cancer Center. Both PC3 and C4-2B cells were maintained in RPMI 1640 medium (Invitrogen) supplemented with 10% fetal bovine serum (Life Technologies) and 1% penicillin/streptomycin. Cell authentication and mycoplasma screening were performed according to institutional guidelines. Cells were transfected using FuGene 6 reagent (Roche) according to the manufacturer’s instructions.

### Plasmids

FKBP51-ARE-luciferase [FKBP51-(−3)], a reporter construct under control of androgen-responsive enhancer of the FKBP51 gene was kindly provided by Dr. Jorma J. Palvimo, University of Eastern Finland^26^,^27^. Expression vectors for the full-length wild-type AR, the T877A and H874Y mutants (kindly provided by Prof. Ian J McEwan), and the AR-V7 isoform were constructed by cloning complementary DNAs (cDNAs) into the p3xFLAG-CMV-14 vector (Sigma-Aldrich, St. Louis, MO).

### Xenograft tumors

MDA-PCa-133-4 (MDA-PCa-133) prostate cancer xenograft tumors were derived from bone lesions of a patient with CRPC as previously described^28^,^29^,^30^,^31^. Written informed consent had been obtained from the patient prior to sample acquisition, according to a protocol approved by the Institutional Review Board of The University of Texas MD Anderson Cancer Center. The xenograft tumors were initially maintained by serial passaging in noncastrated SCID mice. To develop castration-resistant xenograft tumors, 6- to 8-week-old male CB17 SCID mice (Charles River Laboratories International, Inc., Wilmington, MA) were surgically castrated under anesthesia, tumor pieces (about 20 to 30 mm^2^) were implanted subcutaneously in the flank 10 days postcastration, and the mice were monitored weekly for tumor growth. The xenograft tumors were then maintained by serial passaging in the castrate mice.

### Xenograft tumor treatments

To determine the impact of VT-464 and AA treatment, CRPC tumor pieces were implanted subcutaneously in the flank in castrate male SCID mice. The tumors were allowed to grow until they reached about 200 mm^3^. The mice were then randomly divided into three treatment groups (n = 4 or n = 5): control vehicle (0.5% carboxymethylcellulose), VT-464 (100 mg in vehicle/kg body weight/twice daily), and AA (100 mg in vehicle/kg body weight/twice daily). Both drugs were administered by oral gavage daily for 25 days. Tumor dimensions were measured with calipers twice weekly to calculate tumor volume using formula width^2^ × length/2. At day 25, the mice were euthanized, and the tumors were harvested, cut into small pieces, and snap-frozen in liquid nitrogen. The tumor tissues were used to prepare protein lysates for immunoblot analysis and also for steroid profile analysis by mass spectrometry. Tumor volume ratios were calculated by dividing the tumor volume measured on days 4–25 of treatment by the predose tumor volume (day 0). Tumor volume ratios were compared between treatment groups at each time point using a two-tailed, two-sample equal variance Student t-test. P ≤ 0.05 was considered significant. All animal experiments were conducted in accordance with accepted standards of animal care and were approved by the Institutional Animal Care and Use Committee of The University of Texas MD Anderson Cancer Center.

### Patient plasma

Prostate specific antigen (PSA) levels were measured using Beckman Coulter Access Immunoassay System at HS Q Squared Solutions (London, UK). Steroid levels, dehydroepiandrosterone (DHEA), DHEA sulphate, testosterone, androstenedione, progesterone, pregnenolone, cortisol, corticosterone, and ACTH were quantified using triple quadrupole mass spectrometry at Quest Diagnostics-Nichols Institute (San Juan Capistrano, CA). The institutional review board approved this study, which was conducted according to the Declaration of Helsinki, the International Conference on Harmonization, and the Guidelines for Good Clinical Practice. All experimental protocols were approved by the institutional review board of The University of Texas MD Anderson cancer Center and The University of Athens. Written informed consent had been obtained from the patient for treatment and sample acquisition according to the approved protocol.

Further experimental methods are described in detail in Supplementary Materials and Methods.

## Additional Information

**How to cite this article**: Maity, S. N. *et al*. Targeting of CYP17 Lyase by VT-464 Inhibits Adrenal and Intratumoral Androgen Biosynthesis and Tumor Growth of Castration Resistant Prostate Cancer. *Sci. Rep.* **6**, 35354; doi: 10.1038/srep35354 (2016).

## Supplementary Material

Supplementary Information

## Figures and Tables

**Figure 1:**
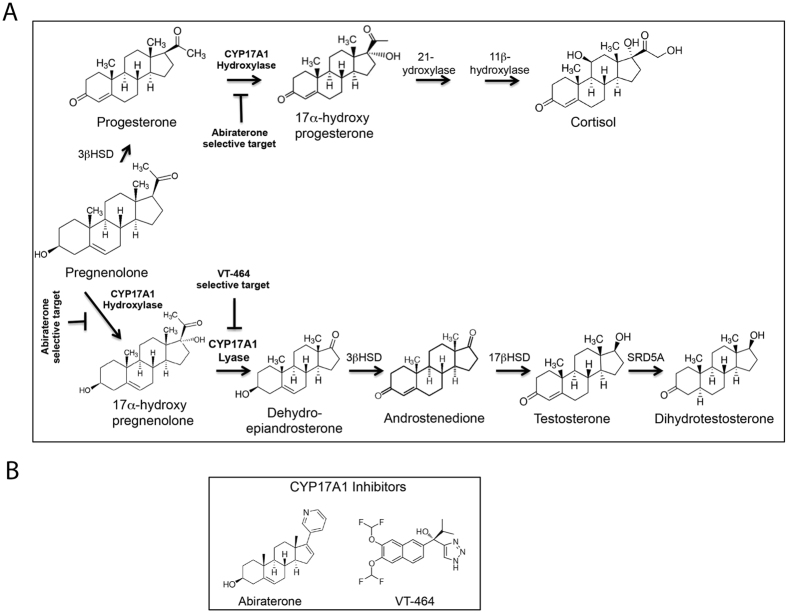
(**A**) Steroid biosynthesis pathway showing chemical conversion by CYP17A1, which is a bi-functional enzyme with hydroxylase and lyase activity that can be selectively inhibited by abiraterone and VT-464. The leydig cells in testis primarily synthesize testosterone, which is converted to potent androgen DHT in prostate. The adrenal gland produces androgen precursor, DHEA, which can also be converted to DHT in prostate. Prostate tumors also contain CYP17A1 and other key steroidogenic enzymes that can drive intratumoral de novo steroid biosynthesis^4^. The CYP17A1 hydroxylase activity mediates adrenal biosynthesis of glucocorticoids, which can be selectively inhibited by abiraterone. In relevance to prostate cancer modeling in mice, it is important to note that in contrast to men, male mice do not produce the adrenal androgen precursor DHEA but produce testosterone by testis^32^,^33^. (**B**) Chemical structures of the nonsteroidal metallophile VT-464 and the steroid-based abiraterone.

**Table 1:** lntratumoral steroid profile measured by mass spectrometry in MDA-PCa-133 tumor tissues treated with VT-464 or AA for 25 days in castrate mice.

MDA-PCa-133 tumor treatments	Mean steroid level in tumor tissue (ng/g ± SEM) n = 4					
	Testosterone			Dihydrotestosterone		
	Mean (SE)	Percent inhibition	P value	Mean (SE)	Percent inhibition	P value
Vehicle	0.23 (0.08)			0.41 (0.11)		
VT-464	0.08 (0.04)	65.2	0.04	0.07 (0.01)	82.9	0.04
AA	0.09 (0.05)	60.8	0.03	0.08 (0.01)	80.4	0.03
