# Supplementary material for: Targeting of CYP17A1 Lyase by VT-464 Inhibits Adrenal and Intratumoral Androgen Biosynthesis and Tumor Growth of Castration Resistant Prostate Cancer
Source: Sci Rep. 2016 Oct 17;6:35354. doi: 10.1038/srep35354 (PMC5066251; doi:10.1038/srep35354)
Supplement: Supplementary Information [file srep35354-s1.pdf]

Targeting of CYP17A1 Lyase by VT-464 Inhibits Adrenal and Intratumoral Androgen Biosynthesis and Tumor Growth of Castration Resistant Prostate Cancer

Sankar N Maity, Mark A Titus, Revekka Gyftaki, Guanglin Wu, Jing-Fang Lu, S Ramachandran, Elsa M Li-Ning-Tapia, Christopher J Logothetis, John C Araujo, and Eleni Efstathiou

Supplementary Materials and Methods

Oligonucleotide primers

The primers for AR and AR-V7 amplification using xenograft total RNAs were hAR1113F, 5'aggatggaagtgcagttagggct3'; hAR3973R, 5'caaggcactgcagaggagtagtgcagag3'; and hAR-V7R, 5'ctgtggatcagctactaccttcagctc3'.

The primers for cloning of AR and AR-V7 in the p3xFLAG-CMV-14 vector were AR-Cla-For, 5'aggctccgaattatcgattaggatggaagtg3'; AR-Xba-Rev, 5'agggtttccaattctagactgggtgaa3'; and ARV7-Xba-Rev-5'caagtcagcctttctagagggtct3'.

The forward and reverse primers for the QRT-PCR assay were as follows: AR, 5'gtcccacttgtgtcaaaagc3' and 5'aaacatggtccctggcagtc3'; AR-V7, 5'atggagctctcacatgtgga3' and 5'agatgcttggaattgccaac3'; PSA, 5'gagagctgtgtcaccatgtg3' and 5'cacaatccgagacaggatga3'; NKX3.1, 5'cagaacgaccagctgagcac3' and 5'agagtccaacagataagacc3'; UBE2C, 5'gtatgatgtcaggaccattc3' and 5'gtgtgttcaagggactatca3'; GAPDH, 5'**cacatcgctcagacaccatg3' and 5'ggcaacaatatccactttaccaga3'.**

Antibodies

The antibodies used in immunoblots experiments are as follows: androgen receptor (N-20), rabbit polyclonal, sc-816 (dilution 1:1000), Santa Cruz Biotechnology; CYP17A1 (cytochrome P450 17A1), rabbit monoclonal, ab125022 (dilution 1:1000), Abcam; AR-V7, mouse monoclonal, AG10008 (dilution 1:100), Precision Antibody; NKX3.1, rabbit polyclonal, 0314 (1:1000), Athena Enzyme System; Flag epitope, mouse monoclonal (M2 clone conjugated to horseradish peroxidase), A8592 (dilution 1:1000), Sigma-Aldrich; GAPDH, rabbit monoclonal (14C10), 2118 (dilution 1:1000), Cell Signaling.

MDA-PCa-133 Xenograft Tumor Tissue

The subcutaneous xenograft tumors were harvested when the tumor size reached 1.5 cm diameter according to institutional guidelines. The tumor tissue were divided for 1) fixation in 10% aqueous formaldehyde solution for paraffin embedding, 2) snap freezing and storage at -80^{0}C, and 3) subsequent implantation. Tissue sections from all formalin-fixed, paraffin-embedded (FFPE) blocks derived from the xenografts were reviewed. Representative blocks were selected and a tissue microarray (TMA) containing 0.6 mm diameter cores was constructed as previously described (1,2)

Immunohistochemistry (IHC) analysis

Tissue sections (4 µm) from the tumors of xenograft, or patient biopsy specimens were subjected to IHC analyses, using an Autostainer Plus (Dako North America, Inc.) as previously published (2). Briefly, serial 4-µm formalin-fixed paraffin-

embedded tissue sections of prostate biopsy or xenograft TMA were deparaffinized by incubation at room temperature in xylene for 5 minutes and then rinsed in 100% ethanol. Antigen retrieval was performed by heating the sections in a Target Retrieval Solution (S1699, Dako, Carpentaria, CA, USA). Incubation with the N-terminal AR antibody (dilution 1:50) (AR441, Dako) or the CYP17A1 antibody (dilution 1:300) (Novus, Littleton, CO, USA) was done at room temperature for 1 hour as previously described (3,4). Slides were then incubated with labeled mouse/rabbit polymer (K4061, Envision Dual Link, Dako) to detect AR, or with labeled anti-goat polymer (ImmPress, Vector, Burlingame, CA) to detect CYP17A1 for 30 minutes at room temperature. Chromogenic detection was performed using 3, 3'-diaminobenzidine (Dab). Slides were counterstained with Mayer's Hematoxylin (Poly Scientific, Bayshore, NY, USA). Tissue sections were viewed with Olympus BX41 Clinical Microscope (Olympus, Center Valley, PA). Specificity of each antibodies were validated in our previous publications (3,4).

Construction of AR and mutant expression plasmids

The DNAs of wild-type AR and AR-V7 open reading frames were PCR amplified using cDNAs of MDA-PCa-180-30 xenografts (2). The amplification was done using HotStart hifidelity polymerase kit (QIAGEN), the amplified DNAs were cloned using the pCR8/GW//TOPO TA Cloning Kit (Life Technologies), and then plasmids from several colonies were sequenced to match wild-type AR and AR-V7. Each cDNA was then amplified using an AR or AR-V7 TOPO plasmid and

primers that introduced ClaI at the 5'-end and XbaI at the 3'-end in order to clone each cDNA into the p3xFLAG-CMV-14 vector and induce expression of each protein as a fusion with the 3xFlag tag. The T877A and H874Y mutants were PCR amplified using cDNAs of C4-2B cells and the MDA-PCa-133 xenograft, respectively, and also expressed as a fusion with the 3xFlag tag.

Transfection and Reporter Assay

Method used for Figure 5A: C4-2B cells were cultured in RPMI1640 medium supplemented with 10% charcoal-stripped fetal bovine serum in 6-well plates at a density of 0.3 x 10^6 cells/well, and transfected with 0.5 μg of the FKBP51-ARE-luciferase plasmid together with 10ng of the TK-renila luciferase plasmid (Promega) for normalization of transfection efficiency using Effectene Transfection reagent (Qiagen) following the manufacturer's instructions. At 16 h after the transfection, the cells were incubated with 1 nM of synthetic androgen R1881 (Sigma-Aldrich) together with various concentration of abiraterone, or VT-464 for 40 h. The Cells were collected after the incubation to measure the luciferase activity using a Dual Luciferase Reporter Assay system (Promega).

Method used for Figure 6 and supplementary Figure 6: PC-3 cells were cultured in RPMI1640 medium supplemented with 10% fetal bovine serum in 6-well plates at a density of 0.3 x 10^6 cells/well, and transfected with 0.25 μg of the FKBP51-ARE-luciferase plasmid, 0.25 μg of the AR or AR-mutants expression vectors, or the vector plasmid control (p3xFLAG-CMV-14), and 10ng of the TK-

renila plasmid using the Effectine Transfection reagent as described earlier. At 16 h after the transfection, the cells were incubated with 1 μM of abiraterone, or 1 μM of VT-464, or 0.5% of DMSO as for vehicle control for 40 h. The cells were collected after the incubation to measure the luciferase activity as described earlier, and to measure expression of AR or AR-mutants proteins by western blot as described below. The relative luciferase activity in each set expressing AR or AR mutants was calculated after subtraction of the basal luciferase activity with the vector plasmid control.

Preparation of Cell or Tissue Protein Extracts and Western Blot Analysis

Whole cell protein extracts were prepared from cultured C4-2B or PC3 cells in a lysis buffer containing 1% Triton x-100, 50 mM HEPES (pH 7.4), 150 mM NaCl, 1.5 mM $MgCl2$, 1 mM EGTA, 10% Glycerol, and protease and phosphatase inhibitors cocktails (Roche). For each million cells, cells were lysed in 150 μl of the lysis buffer for 30 min in ice, centrifuged for 5 min at 10000 rpm at 4 ^{o}C, and the supernatants were used for western blot analysis as shown in Figures 4 and 5.

Extracts from flash-frozen xenograft tumor tissue was prepared using T-PER tissue protein extraction regent (Thermo Fisher Scientific, Waltham, MA, USA) supplemented with protease and phosphatase inhibitors cocktails (Roche). Frozen tumor tissues were grinded with mortar and pestle, incubated with the extraction buffer (2 ml of buffer per 0.1 g of tissue) in ice for 30 min, sonicated for 10 sec for 3 times in ice, centrifuged at 12,000 rpm for 5 min at 4 ^{o}C, and then

the supernatant was collected and used for western blot analysis as shown supplemental Figure 6C.

Cell or tissue lysates (40 μg of protein) were separated on 4%–20% Tris-glycine polyacrylamide gels, transferred to nitrocellulose membranes (Invitrogen, Carlsbad, CA) and incubated overnight at 4°C with the primary antibodies described above. Next, the mixtures were incubated for 1 h at room temperature with horseradish peroxidase-conjugated anti-mouse or anti-rabbit antibody (Cell Signaling) at a 1:3,000 dilution. Immunoblots were developed by using enhanced chemiluminescence (Amersham GE Healthcare Bio-Sciences Corp. Piscataway, NJ).

RNA and QRT-PCR analysis

Total RNA was extracted from cell lines using the RNeasy Mini Kit (Qiagen). The relative mRNA level for each gene was quantified by using QRT-PCR with SYBR Green (Applied Biosystems, Inc., Foster City, CA) and the primers described above. The mRNA level for each gene was calculated from the values in the linear range of the PCR cycles and normalized to the value for the reference mRNA of *GAPDH* and *ACTB* (2) as follows: ΔC_T = average C_T of reference − average C_T of the specific gene, where C_T is the threshold cycle. The relative level of expression of each gene with respect to the reference gene (*GAPDH* or *ACTB*) was then calculated as $2^{\Delta CT}$. For each gene, data from three

independent measurements were used to calculate means and standard deviations.

Measurements of PSA and testosterone in mouse serum

Mouse blood was collected by either mandibular venipuncture or cardiac puncture in an Eppendorf centrifuge tube, kept on the lab bench for 30 min at room temperature, and centrifuged at 3,000 rpm for 5 min at room temperature, and then the supernatant (serum) was collected for analysis. Serum PSA and testosterone were measured by ELISA using kits from American Qualex, San Clemente, CA (catalog no. KD4310), and Alpha Diagnostic International, San Antonio, TX (catalog no. 1880), respectively, following the protocols provided in each kit.

Liquid chromatography–triple quadrupole mass spectrometry steroid analysis for MDA-PCa-133 patient-derived xenograft. Stable-isotope-labeled steroid internal standards progesterone-2,3,4-$^{13}C_3$, cortisol-9,11,12-$^{2}H_4$, dihydrotestosterone-2,3,4-$^{13}C_3$, and testosterone-2,3,4-$^{13}C_3$ were purchased from IsoSciences (King of Prussia, PA). Pure-standard cortisol, progesterone, dihydrotestosterone, and testosterone were purchased from Steraloids. Hydroxylamine hydrochloride and ultrapure methanol and water (Chromasolv) were purchased from Sigma-Aldrich. Steroid internal standards progesterone-$^{13}C_3$, dihydrotestosterone-$^{13}C_3$, cortisol-$^{2}H_4$, and testosterone-$^{13}C_3$ (0.5 ng each) were added to C4-2B, PC3, and MDA-PCa-133 homogenates. Steroids were extracted using tert-butyl methyl ether, and the separated organic layer was

evaporated under nitrogen. The extracts were subsequently derivatized using hydroxylamine hydrochloride in water/methanol (5). An Agilent (Santa Clara, CA) 6490 triple quadrupole mass spectrometer equipped with a Jet Stream electrospray ion source(Agilent), a 1290 Infinity ultrahigh-performance liquid chromatography system (Agilent) and MassHunter Workstation software (Agilent) was used to quantify steroids. Chromatographic separation of steroid oximes was conducted with a Chromolith C_{18} reverse phase column (50 x 2 mm) with a matching Chromolith guard column (5 x 2 mm) using a mobile phase gradient from 30% methanol–water with 0.1% formic acid to 95% methanol–water with 0.1% formic acid. Steroid oximes were introduced into the electrospray ion source and analyzed in the positive ion mode. Molecular ions for cortisol (*m/z* 393.3), progesterone (*m/z* 345.2), DHT (*m/z* 306.2), and testosterone (*m/z* 304.2) and for the $^{13}C_3$ internal-standards cortisol (*m/z* 397.4), progesterone (*m/z* 348.2), DHT (*m/z* 309.2), and testosterone (*m/z* 307.2) were selected in the first quadrupole and quantified using product ions *m/z* 145.1 for cortisol, *m/z* 124.2 for progesterone, *m/z* 255.2 for DHT, *m/z* 124.1 for testosterone and for internal-standard cortisol (*m/z* 148.2), progesterone (*m/z* 127.2), DHT (*m/z* 258.3), and testosterone (*m/z* 127.1). The lower limit of quantification was 2.5 femtograms for testosterone and progesterone and 25 femtograms for cortisol and dihydrotestosterone.

References

1. Efstathiou E, Karlou M, Wen S, Hoang A, Pettaway CA, Pisters LL, et al. Integrated Hedgehog signaling is induced following castration in human and murine prostate cancers. Prostate 2013;73(2):153-61.

2. Tzelepi V, Zhang J, Lu JF, Kleb B, Wu G, Wan X, et al. Modeling a lethal prostate cancer variant with small-cell carcinoma features. Clin Cancer Res 2012;18(3):666-77.
3. Efstathiou E, Titus M, Tsavachidou D, Tzelepi V, Wen S, Hoang A, et al. Effects of abiraterone acetate on androgen signaling in castrate-resistant prostate cancer in bone. J Clin Oncol 2012;30(6):637-43.
4. Efstathiou E, Titus M, Wen S, Hoang A, Karlou M, Ashe R, et al. Molecular characterization of enzalutamide-treated bone metastatic castration-resistant prostate cancer. Eur Urol 2015;67(1):53-60.
5. Keski-Rahkonen P, Huhtinen K, Poutanen M, Auriola S. Fast and sensitive liquid chromatography-mass spectrometry assay for seven androgenic and progestagenic steroids in human serum. J Steroid Biochem Mol Biol 2011;127(3-5):396-404.

Supplementary Figure Legends

Supplementary Figure 1. Level of Testosterone, and Androstenedione in patient plasma during treatment with VT-464 collected each month for 40 months

Supplementary Figure 2. Level of Cortisol, Corticosterone, Progesterone, and ACTH in patient plasma during treatment with VT-464 collected each month for 40 months. Blood was drawn from patient during morning between 9 and 10 AM.

Supplementary Figure 3. Development and characterization of castration-resistant MDA-PCa-133 patient-derived xenograft (PDX) tumors in male SCID mice. (A) Strategy to develop PDX tumors in male SCID mice. (B)

Representative images of hematoxylin-eosin (H&E) staining of MDA-PCa-133 tumors showing adenocarcinoma. The tumors samples in this analysis were prepared from the harvested tumors when it reached 1.5 cm diameter in castrated and non-castrated (intact) SCID mice. Original magnification, x200. (C) Comparison of MDA-PCa-133 tumor growth rate in castrated and non-castrated SCID mice. The tumor volumes were measured with a caliper twice a week for 4 weeks. The estimated tumor volume (V) was calculated by the following formula $V = W^2 \times L \times 0.5$, where W represents the largest tumor diameter in centimeters and L represents the next largest tumor diameter. Growth rate in each group (number of mice = 4) was calculated as mean tumor volume increase in cubic centimeter per day (CM3/day) +/- SD. (D) and (E) Comparison of serum testosterone and PSA in castrated and non-castrated SCID mice bearing subcutaneous tumors. Mouse blood was collected at the time of tumors harvest when the MDA-PCa-133 tumors reached 1.5 cm diameter, and was used to prepare serum to measure testosterone (D) and PSA (E) by ELISA. The results are expressed as the mean value +/- SD (number of mice = 4 in each group).

Supplementary Figure 4. Induction of AR and AR-V7 in MDA-PCa-133 PDX tumors in castrated mice. (A) Locations of primers used to detect AR and AR-V7 by quantitative real-time PCR. (B) Relative mRNA levels of AR and AR-V7 in the MDA-PCa-133 tumors in castrated and non-castrated SCID mice. (C) Protein extracts from tumor tissues were analyzed by immunoblot to detect AR, CYP17, and GAPDH (control). The three gels were run under same experimental conditions. Both AR and AR-V7 were detected by N-terminal AR antibody.

Individual immunoblots are included at end of the supplementary figures. (D) Representative images of immunohistochemistry analysis of androgen receptor (AR) expression in the xenograft tumors detected by N-terminal AR antibody showing diffuse nuclear expression of AR. Original magnification, x200. The tumor samples were collected similarly as described in supplementary figure 3.

Supplementary Figure 5. Boxplot analysis of intratumoral level of progesterone and cortisol in MDA-PCa-133 tumor treated with VT-464 and AA. The steroid level data in supplementary table 2 was used for the boxplot.

Supplementary Figure 6. Effects of the AR-V7 isoform on FKBP51-ARE reporter in PC3 cells treated with vehicle, Abi, or VT-464. (A) AR-V7 was expressed as 3x-Flag epitope tag. (B) AR-V7 expression was monitored by Western blot with anti-Flag antibody. (C) FKBP-51-ARE reporter activity. DMSO, dimethyl sulfoxide.

VT-464 patient:
Levels of PSA, Testosterone and Androstenedione

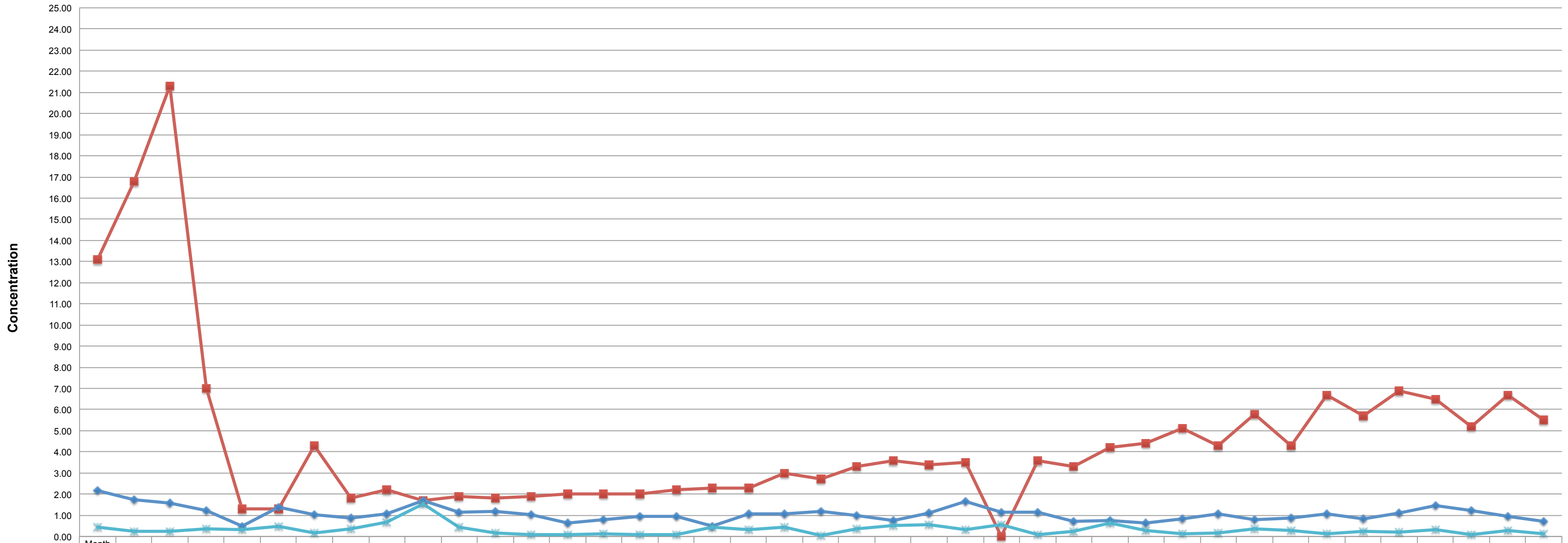

	Month Baseline	1	2	3	4	5	6	7	8	9	10	11	12	13	14	15	16	17	18	19	20
PSA microgram/L	13.10	16.80	21.30	7.00	1.30	1.30	4.30	1.80	2.20	1.70	1.90	1.80	1.90	2.00	2.00	2.00	2.20	2.30	2.30	3.00	2.70
Androstenedione nmol/L	2.16	1.75	1.57	1.24	0.49	1.40	1.01	0.87	1.05	1.71	1.15	1.19	1.01	0.63	0.80	0.94	0.94	0.49	1.08	1.05	1.19
Testosterone nmol/L	0.42	0.24	0.24	0.35	0.31	0.49	0.17	0.35	0.69	1.53	0.45	0.17	0.07	0.07	0.14	0.10	0.10	0.42	0.31	0.45	0.03

	21	22	23	24	25	26	27	28	29	30	31	32	33	34	35	36	37	38	39	40
PSA microgram/L	3.30	3.60	3.40	3.50	0.00	3.60	3.30	4.20	4.40	5.10	4.30	5.80	4.30	6.70	5.70	6.90	6.50	5.20	6.70	5.50
Androstenedione nmol/L	0.98	0.77	1.12	1.64	1.15	1.15	0.73	0.77	0.63	0.84	1.08	0.80	0.87	1.08	0.84	1.12	1.47	1.22	0.94	0.70
Testosterone nmol/L	0.38	0.52	0.56	0.31	0.56	0.07	0.24	0.62	0.28	0.14	0.17	0.38	0.28	0.14	0.24	0.21	0.31	0.10	0.28	0.14

Maity et al.
Supplementary Fig 1

VT-464 patient: Levels of Cortisol, PSA, Corticosterone, Progesterone and ACTH

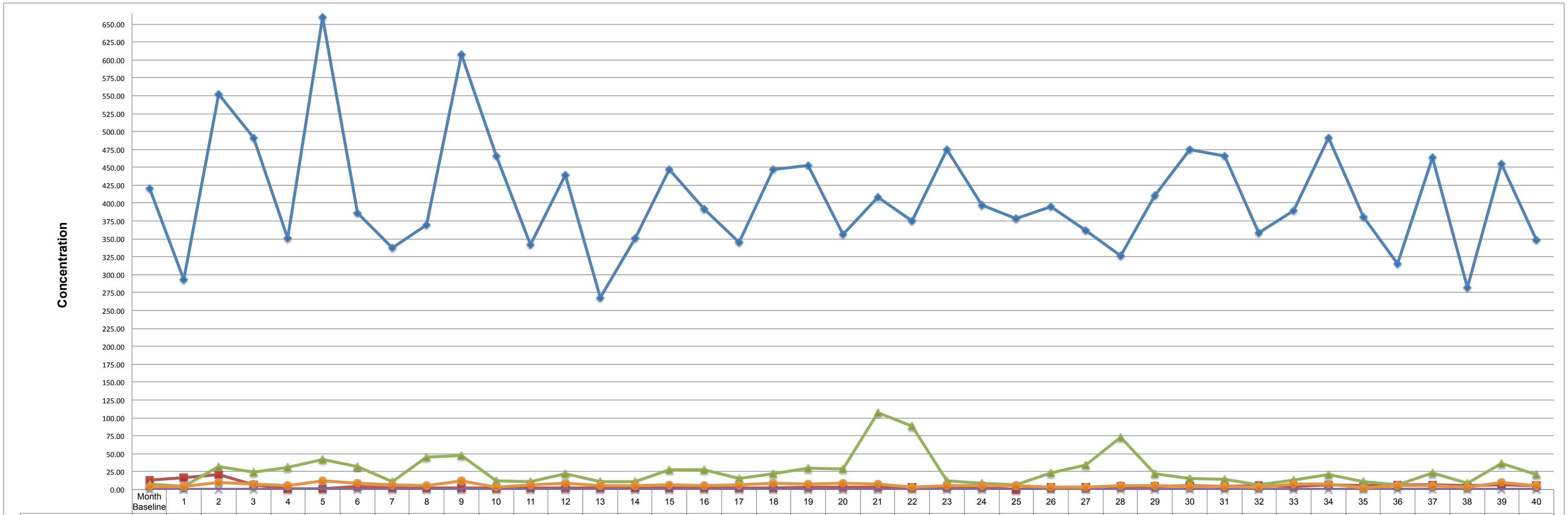

Month	Baseline	1	2	3	4	5	6	7	8	9	10	11	12	13	14	15	16	17	18	19
PSA microgram/L	13.10	16.80	21.30	7.00	1.30	1.30	4.30	1.80	2.20	1.70	1.90	1.80	1.90	2.00	2.00	2.00	2.20	2.30	2.30	3.00
Cortisol nmol/L	420.00	293.00	552.00	491.00	351.00	660.00	386.00	337.00	370.00	607.00	466.00	342.00	439.00	268.00	351.00	447.00	392.00	345.00	447.00	453.00
Cortiscosterone nmol/L	7.66	4.36	32.02	24.45	30.98	42.05	32.02	10.49	45.37	47.83	12.20	10.78	22.48	11.42	11.10	27.95	27.95	15.58	22.60	30.11
Progesterone nmol/L	0.30	0.30	0.30	0.30	0.30	0.60	0.30	0.30	0.30	0.30	0.30	0.30	0.30	0.30	0.30	0.30	0.30	0.30	0.30	0.30
ACTH pmol/L	4.40	3.80	9.50	8.00	5.30	11.80	8.90	6.90	5.30	11.80	3.60	6.90	9.30	5.60	5.30	6.70	5.60	6.40	8.70	7.50

Month	20	21	22	23	24	25	26	27	28	29	30	31	32	33	34	35	36	37	38	39	40
PSA microgram/L	2.70	3.30	3.60	3.40	3.50	0.00	3.60	3.30	4.20	4.40	5.10	4.30	5.80	4.30	6.70	5.70	6.90	6.50	5.20	6.70	5.50
Cortisol nmol/L	356.00	408.00	375.00	475.00	397.00	378.00	395.00	362.00	326.00	411.00	475.00	466.00	359.00	389.00	491.00	381.00	315.00	464.00	282.00	455.00	348.00
Cortiscosterone nmol/L	29.22	106.76	87.97	12.43	8.55	6.88	23.03	34.28	72.74	22.51	15.66	14.54	6.73	13.61	20.40	11.18	6.76	23.09	9.13	36.15	20.55
Progesterone nmol/L	0.30	0.30	0.30	0.30	0.30	0.30	0.30	0.30	0.30	0.30	0.30	0.30	0.30	0.30	0.30	0.30	0.30	0.30	0.30	0.30	0.30
ACTH pmol/L	8.70	7.80	3.10	6.00	5.10	5.30	2.90	2.90	5.60	4.90	4.00	3.80	3.60	7.30	7.50	2.40	5.10	5.30	3.60	10.40	6.00

Maity et al.
Supplementary Fig 2

MDA-PCa-133 (Patient Derived Xenograft)

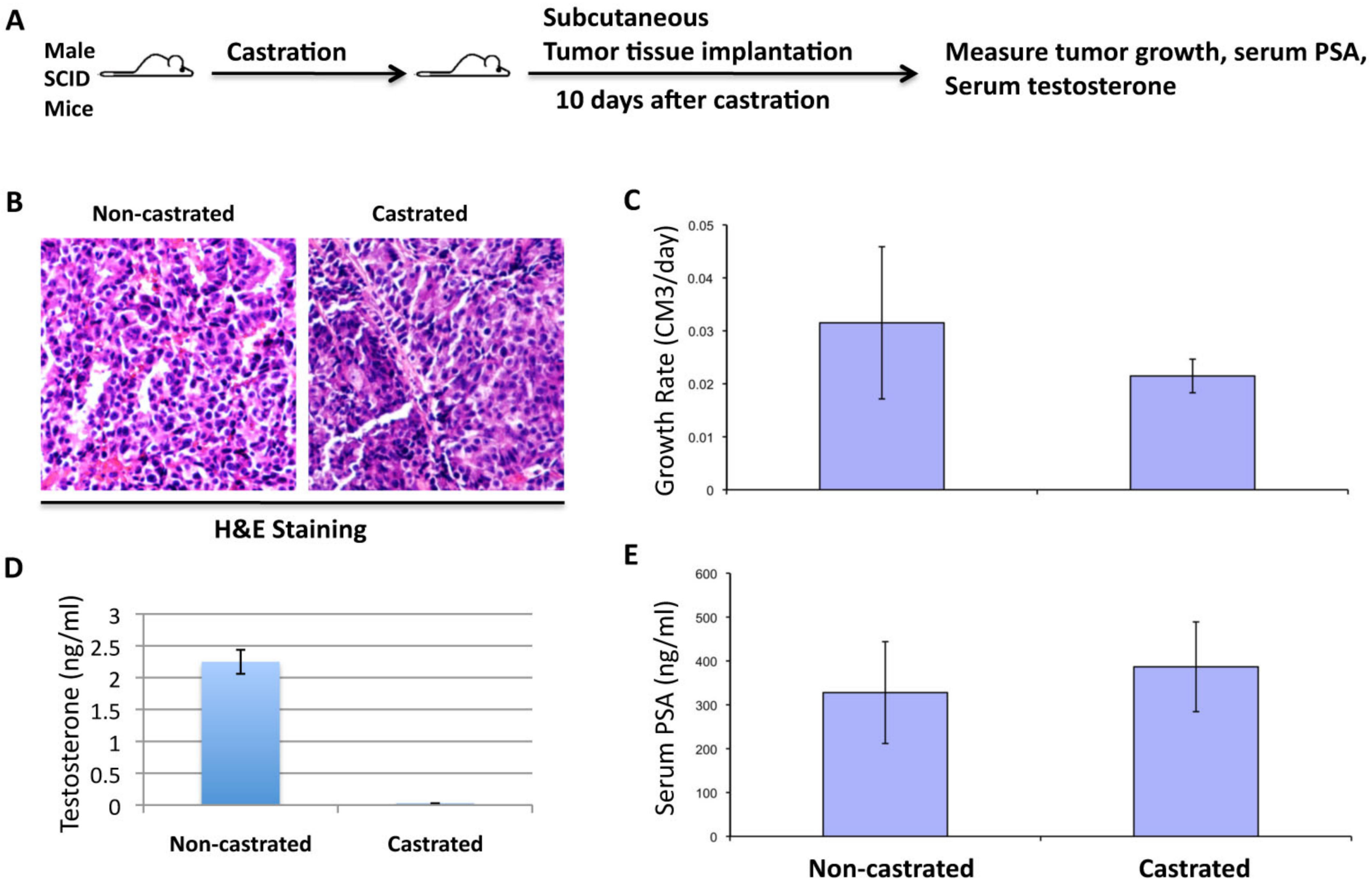

Maity et al.
Supplementary Fig 3

MDA-PCa-133

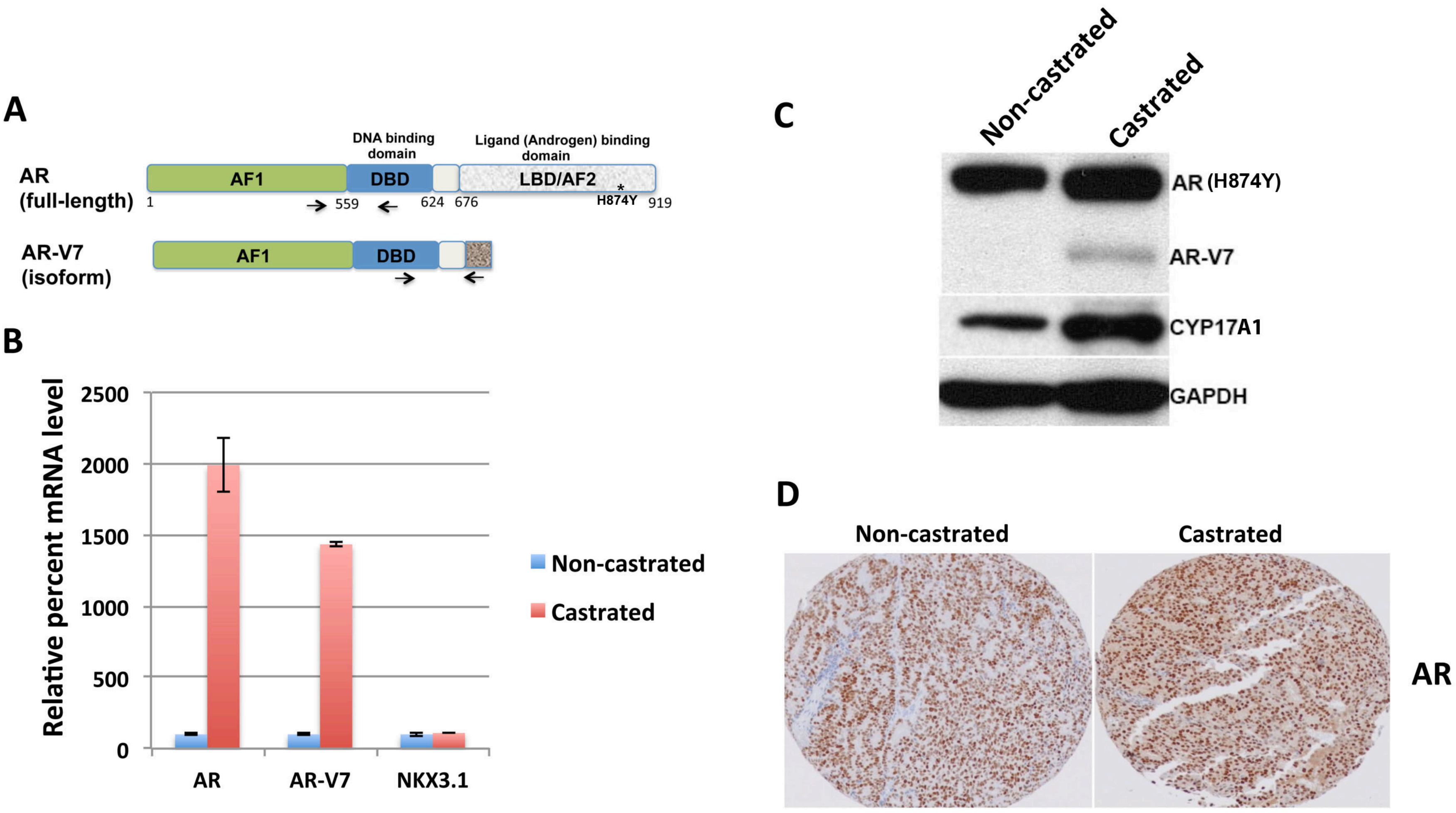

Maity et al.
Supplementary Fig 4

Supplementary Table 1: Testosterone levels in pooled MDA-PCa-133 xenograft tumors (n = 3) and livers (n = 3) from tumor bearing mice with or without castration. The samples were injected 3 times to obtain 3 measurements to calculate SD.

Tissue	Mean Testosterone (ng/g of tissue +/- SD)
Xenograft tumor (non-castrate)	0.02 +/- 0.007
Xenograft tumor (castrate)	0.1 +/- 0.02
Liver (non-castrate)	0.01 +/- 0.004
Liver (castrate)	<0.001 +/- 0.001

Maity et al.

Supplementary Table 2: Intratumoral profile of progesterone and cortisol in MDA-PCa-133 tumor tissue treated with VT-464 and AA

**MDA-PCa-133 Tumor Treatments**	**Mean Steroid level in tumor tissue (ng/g +/- SEM, n=3)**					
	Progesterone			Cortisol		
	Mean (SE)	Percent Increase	P Value	Mean (SE)	Percent Inhibition	P Value
Vehicle	0.14 (0.03)			0.64 (0.07)		
VT-464	0.32 (0.21)	228	0.25	0.41 (0.08)	35.9	0.05
AA	0.51 (0.41)	364	0.23	0.02 (0.01)	96.8	<0.001

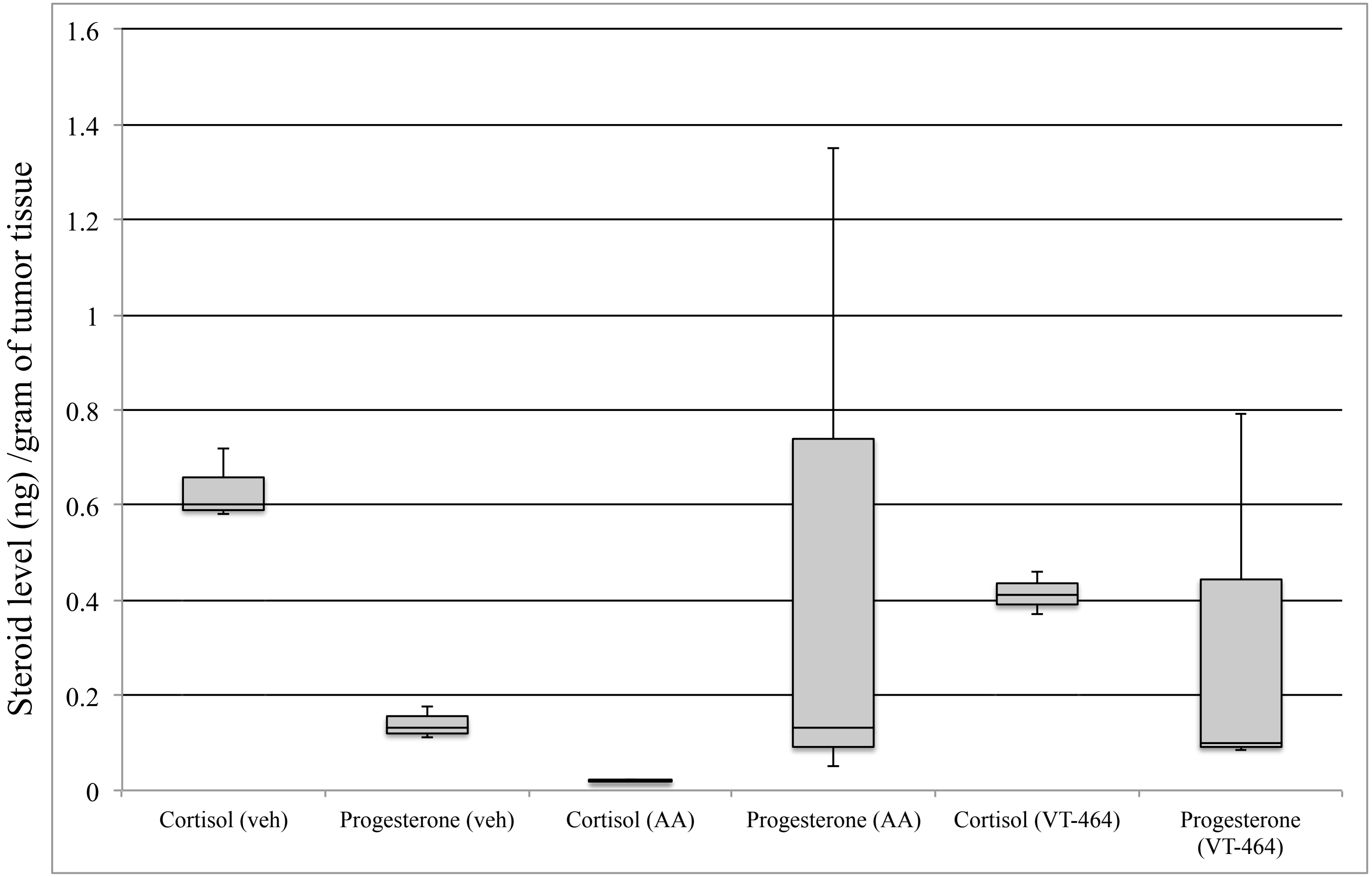

Maity et al.
Supplementary Fig 5

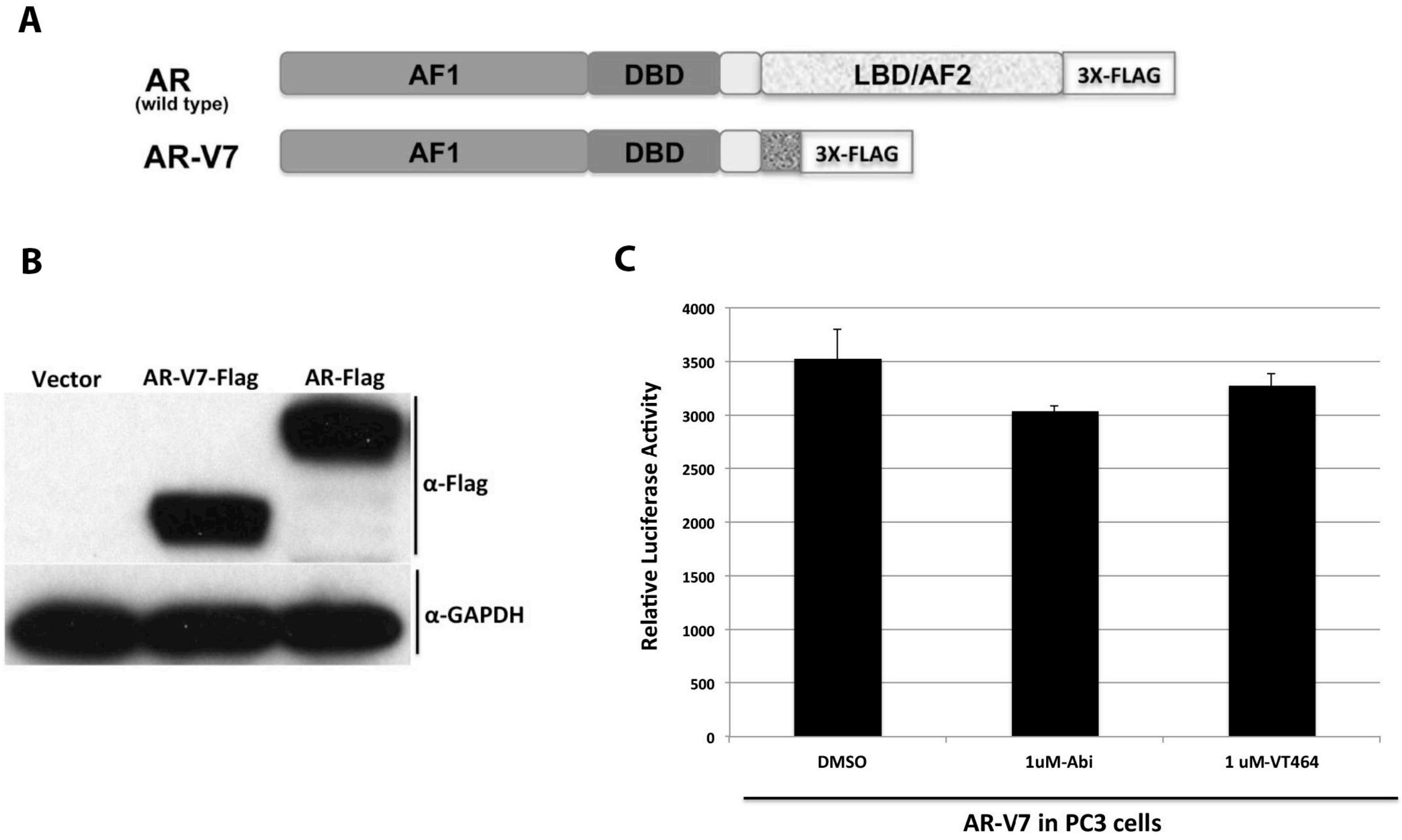

Maity et al.
Supplementary Fig 6

Immunoblots of Figure 5B

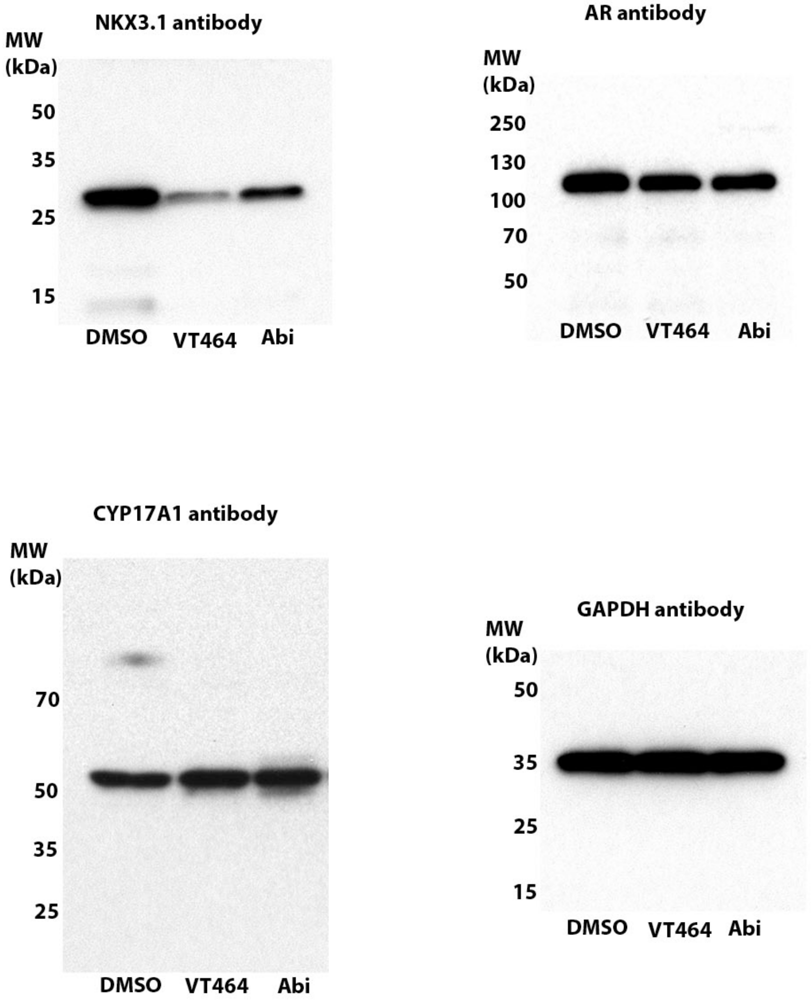

Immunoblots of Supplementary Figure 4C

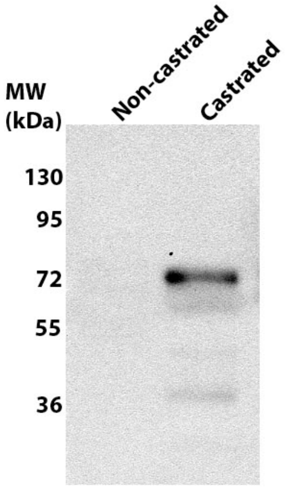

AR-V7 antibody

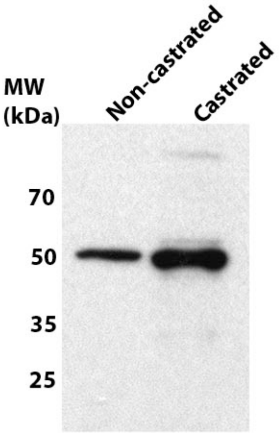

CYP17A1 antibody

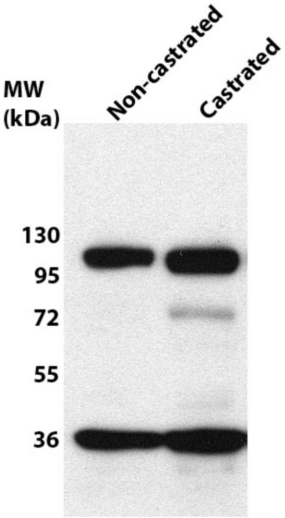

AR and GAPDH antibodies
